# Yellow Fever in Mexico

**Published:** 1848-05

**Authors:** B. Rush Mitchell

**Affiliations:** U. S. N.


### *Yellow Fever in Mexico.* By B. Rush Mitchell, M. D., U. S. N.

For the following valuable contribution we are indebted to Professor T. D. Mitchell, of Lexington, Ky., father of the writer.

*U. S. Steamer Spitfire.*
Tlacotalpam, March 22d, 1848.

Dear Father,—As you desire to know what medical items of interest I may have picked up in the Gulf, I write this letter wholly upon medical subjects. The most alarming disease with which we have had to contend in the squadron, was yellow fever; and that we experienced it in a most virulent and epidemic manner last summer, the history of the squadron most conclusively establishes. Of twelve medical officers upon the station, four died within ten days of each other, among them being some who were inured to the climate; indeed, acclimation seemed to be no shield whatever, for the most virulent cases were those of men who were in their *second* summer in the climate. It may be that owing to our vessels being wholly at sea the previous summer, the men may not have been subject to acclimatizing influences; but as they were rarely out of sight of Vera Cruz, this is hardly probable.

The immediate cause of the general outbreak of yellow fever in the squadron was, without doubt, the expedition last made to Tobasco. This town and vicinity is notoriously a hot bed of disease; and as the expedition was had in the month of June, and some eight hundred sailors were marched in the hot sun, dragging cannon through sand for some ten miles, and afterwards exposed in Tobasco to rain, dew, and bad quarters, it is not to be wondered that epidemic disease soon sprang up. True, some sporadic cases of yellow fever had occurred prior to the expedition; but as the parties were in ill ventilated and dirty ships, and had exposed themselves to solar influence, it was only what we had a right to expect. During the few days the expedition remained in Tobasco, it became necessary to establish a hospital; cases of bilious and congestive fevers, and dysentery, accumulating so fast, as to imperiously demand sick quarters. The expedition returned to Anton Lizardo anchorage, fourteen miles from Vera Cruz, on the

30th of June. On the 4th of July another expedition of some hundred marines, etc., was made up this river to Casamaloapam; and on its return, I treated a genuine case of yellow fever on board the steamer Petrita, a vessel old, and in a very foul condition.

About the 7th of July, yellow fever opened the dance of death on board the steamer Mississippi, one of the vessels attached to the Tobasco expedition, and so rapidly did it spread, that in three or four days two hundred men were in Salmadina Hospital, and not enough well men left on board the vessel to weigh anchor. For a time the disease seemed to confine itself to the Mississippi, and its virulence was doubtless aided by a spontaneous combustion of her coal, which, besides requiring the extra working of the men, filled the ship with a foul atmosphere. The frigate Raritan, which left Tobasco *direct* for the United States, had the yellow fever to break out a few days after her departure, and lost some twenty odd men and officers before arriving at Norfolk. Her dirty condition was a matter of notoriety.

The Commodore, perceiving the continual increase of the sickness on board the Mississippi, resolved to send her to Pensacola; and she accordingly left, August 11th, carrying some three hundred sick. Prior to her departure, yellow fever appeared on board the sloop of war Decatur, the steamers Spitfire and Vixen, and the store ship Fredonia. About the 22d of August, the store ship Electra arrived from Pensacola. When she left that place, yellow fever was very prevalent. She brought out, as an after examination developed, bad stores, and was otherwise in a foul state. Four days after her arrival, the greater part of her officers and crew were in the hospital of Salmadina; and on August 29th, Dr. Kearney, fleet surgeon, who had come out as a passenger in the Electra, died from yellow fever, in the hospital. Dr. Bates of the Vixen died the previous day; and Dr. J. H. Smith, of the Spitfire, sickened August 31st, and died September 6th. He had left his vessel some ten days before for hospital duty: he had been at Tobasco, as had also Dr. Hastings, and these were the *only* two cases of yellow fever that originated, or in whom the symptoms were first developed on the island. On the 30th of August, I was ordered up to assume the medical charge of the U. S. sloop Germantown; but on my arrival the next day at Anton, finding that the medical officer had come off from the hospital, convalescing, and that Dr. Smith was sick, I was ordered to the hospital.

On going ashore, I found one hundred and forty patients in the hospital, and Dr. Smith in so precarious a state, as to forbid the hope that he would survive. In addition to the hospital buildings, we had four tents erected for the accommodation of the convalescing, as well as for the reception of new patients, the average reception of whom for weeks was ten or twelve daily. The brig Stromboli, too, arrived with a new class of cases, most virulent bilious intermittents, and in short *every* vessel, including the store ship Relief, had more or less of yellow fever on board. And thus it continued until October 1st, when I received into the hospital the last case of epidemic yellow fever for the season, from the Fredonia, a man, who died shortly after his admission. We had, it is true, several admissions of yellow fever up to November 1st, but the epidemic visitation and violence ended October 1st. About the 12th of September, my colleague, Dr. Hastings took the fever, and went to Vera Cruz, his proximity to where his friend Dr. Smith had so recently died being prejudical to his recovery; and this left me quite alone, surrounded by from one hundred and twenty to one hundred and forty patients; and thus I remained with a single exception, until relieved by Dr. Spotswood, November 10th.

Such is a history of the beginning, progress, and end of the yellow fever in the Home Squadron, during last summer. What caused its extensive prevalence? Upon this point there was some difference of opinion, some of the older medical officers alleging, that it was because of exhalations from the coral reefs of the anchorage, which were daily exposed at low tide. But that this, however it may have abetted the disease was not its cause, is evident from these facts, first, that the Raritan had it on board directly after leaving Tobasco, and without coming to Anton Lizardo; second, that the disease broke out on the Electra within two days after her arrival at Anton, as it did also on the Relief; third, that the Fredonia was among the latest, if not the last vessel on board which the disease appeared, (and then among marines who had been at Tobasco,) and did not manifest much violence, until the crew were worked in the sun preparing the ship for sea; and yet, this vessel had been lying the whole summer exposed to prevailed on board the shipping at Alvarado, two miles from the sea, and twenty from any coral reefs; fifth, that but *two* cases of yellow fever originated or seemed to originate on Salmadina island, the centre of the anchorage; and this, when there were at least forty persons there constantly, who never had had the fever; and lastly, the disease did *not* prevail on board the shipping laden with coal at the anchorage, although there were six vessels which had been there since March. These facts I deem sufficient to establish the groundlessness of the opinion I have adverted to, and to prove that we must seek elsewhere for the cause of yellow fever in the home squadron.

My opinion then is this, that although sporadic cases of yellow fever might, and probably would have occurred in the squadron, if they had remained at Anton Lizardo, yet, that there would have been nothing like the epidemic visitation we experienced, *if the expedition to Tobasco had not been made.* In coming to this opinion I design to cast no blame upon the Commodore, for it was impossible, *a priori*, to predict that such results would flow from the expedition. But that the expedition had such results, I think evident from the history of the disease. Although yellow fever existed epidemically in Vera Cruz prior to the departure of the Tobasco expedition, but four or six cases had been noticed in the whole squadron. Immediately upon its return to the anchorage, however, it began to show its head, and in a few days two or three hundred were attacked. Am I jumping to conclusions? The coincidence is, at least, strong in behalf of my view. Another strong fact is, that the United States' schooner Flirt, which was at the anchorage during the first half of the epidemic, did not have the disease on board; nor did the Saratoga, until after an exposure of one month to the infected atmosphere of Vera Cruz. The facts then I conceive to be these, that the exposure of the men to solar influence, fatigue, rain, etc., at Tobasco, and their subsequent return to confined and sometimes dirty vessels, and exposure on board, originated and propagated the disease in question. All who were at Tobasco were exposed to malarious influence, which only needed to be aided by sea air to develope yellow fever.

Upon this point, the general origin of yellow fever, I hold this opinion, which I claim as *original,* that yellow fever cannot exist epidemically, nor be originated without the presence of *sea air.* Marsh miasmata alone and unaided may, and does produce fevers of various grades; but unless the individual be exposed to a sea air, he cannot have yellow fever. I have not the works of Dr. Rush by me, but if my recollection serves me, you will find that in the Philadelphia epidemics, the prevailing winds, either before or during the epidemics, *were from the sea;* thus impregnating the Philadelphia atmosphere with sea air. You know that the range from the sea coast of yellow fever is limited, and when it extends beyond those limits, if you enquire of the winds, they will tell you why it is. This town is twelve miles from the Gulf, but is separated from it by a chain of hills and mountains; it never has yellow fever, while Alvarado, ten miles nearer the sea and exposed to sea breezes, has. Yet this town is abundant in malaria; and Alvarado is built on sand. And such is the general history of these parts. The island of Salmadina is a mass of sand, and no malaria is there generated. It is moreover six miles to windward of land; yet but two cases of yellow fever occurred among the island residents, and these residents but recently removed from their ships. Why was this? Was it not because there was no marsh miasmata there to mix with the sea air? If not, why did the vessels but three hundred yards from the island suffer so severely? Was it not because their vegetable and animal exhalations afforded a something which, united with sea air, developed yellow fever.

But speculations apart, I come to something more tangible. With the symptoms of yellow fever and its progress all are familiar, and it is therefore on its treatment and lesions that I design only to remark. Death in yellow fever is caused either by extensive brainial disease, or abdominal affection, and consequently our care is to guard against disease attaining a mortal inroad in these localities. I never use general depletion for either determinations of the disease; both in the hospital of Salmadina and that of Vera Cruz, such a course eventuated in harm and only harm. When the head symptoms ran high, I bled locally by cups, and blistered; when the gastric symptoms were most prominent, I blistered the epigastrium. In either case, my main reliance was in the production of speedy and copious bilious purgation, to attain which, I immediately administered calomel, ℈iss. pulv. jalap. comp. grs. xl.; if no operation was had in four hours, repeated the dose. By this I obtained free discharges from the bowels, liver, the influence of the coral reef atmosphere; fourth, that the disease and kidneys, and had the pleasure to find the intense pain of the lumbar region—so distressing a part of the disease—almost immediately relieved. Simple jalap failed to answer the same beneficial purpose with the compound powder.

For the nausea and vomiting attendant upon the disease, an etherial tincture of kreasote was almost invariably beneficial. By it I arrested the black vomit itself, and procured recovery. The prescription usually employed was,

| | |
|---|---|
| Kreasote | gtt. xx. |
| Eth. sulph. | ℥i. |
| Spts. lavend. comp. | ℥i. |

M. A teaspoonful every fifteen minutes until the nausea or vomiting ceased.

From three to four doses in ordinary, and six to ten in malignant cases, never failed in my hands to achieve the end designed. But to insure its success as a remedy, as well as to save the life of the patient, it is necessary to deny the use of any liquid aliment or drink to the stomach. So great was the irritability in a majority of cases, that even the entrance of water from ice melted in the mouth, was not tolerated five minutes by that organ, ejection almost immediately ensuing.

Such is an outline of the course of treatment I pursued in the yellow fever of the squadron, and with great success. I combatted *symptoms* as they arose, meeting them as they appeared, boldly, and vanquished necessarily the cause of their appearance. One word as to contagion. No medical officer who was here last summer, that I know of, believes in it for a moment, nor did a single item of proof that it caused a single case here develope itself. In treating the disease, the navy went ahead of the army, we losing about *one in twenty-seven*, the army one in three or four.

To show you in what particular parts the disease spent its virulence, I append a description of the lesions I found on post mortem. They were referable to two general classes, cranial and abdominal.

Those cases in which head symptoms, such as fierce delirium or profound coma, were strongly marked from the first, evidenced little or no lesions in the abdomen, and vice versa. The head lesions were, effusion into all the ventricles of the brain in large amount, and so softened a brain as to have little more consistence than jelly. About the medulla oblongata, softening was very evident, accompanied with much redness of the upper part of the spinal cord.

In the abdomen, the most prominent changes were those in the liver, spleen, and kidneys. The liver was generally soft in texture, and its colour of an *olive green*,—the gall bladder being distended with black tarry matter—a similar semi-fluid being found in the stomach; the spleen was usually the consistence of clotted blood and much enlarged; the kidneys were in all cases more or less inflamed, especially on their periphery; in some cases considerable cystitis existed. These are all the lesions that were observed worthy of note.

