# Correction

**Published:** 1848-06

**Authors:** 


### CORRECTION.

It is seldom that we trouble our readers with corrections of either our own errors or those of the printer, after publication; but in the last number, so serious a blunder occurs in Dr. Mitchell's article on yellow fever, as to render it absolutely unintelligible. In "making up," the printer transferred a line from the bottom of page 287 to the top of page 290, thus destroying the connection of the pages.

